# Prevalence of mild cognitive impairment and its association with handgrip strength in patients on hemodialysis

**DOI:** 10.1038/s41598-022-07550-4

**Published:** 2022-03-09

**Authors:** Sumi Hidaka, Akinori Nishimiura, Masahiro Hirata, Kunihiro Ishioka, Takayasu Ohtake, Machiko Oka, Teiichi Tamura, Kazuhiko Shibata, Masahiro Nishihara, Tadashi Kuji, Jin Oshikawa, Hidehisa Satta, Kiyotaka Imoto, Takehiko Kunieda, Kiyoshi Ozawa, Shuzo Kobayashi

**Affiliations:** 1 Kidney Disease and Transplant Center, Shonan Kamakura General Hospital, Okamoto 1370-1, Kamakura, 247-8533 Japan; 2 Rehabilitation Unit, Shonan Kamakura General Hospital, Kamakura, Japan; 3 Center for Clinical and Translational Science, Shonan Kamakura General Hospital, Kamakura, Japan; 4 Department of Nephrology, Shonan Fujisawa Tokushukai Hospital, Fujisawa, Japan; 5 Yokosuka Clinic, Yokosuka, Japan; 6 Yokohama Minami Clinic, Yokohama, Japan; 7 Tojin Clinic, Yokohama, Japan; 8 Yokodai Central Clinic, Yokohama, Japan; 9 Department of Nephrology, Yokohama Sakae Kyosai Hospital, Yokohama, Japan; 10 Kasama Clinic, Yokohama, Japan; 11 Shonan Clinic, Kamakura, Japan

**Keywords:** Nephrology, Neurology

## Abstract

Dementia is associated with a high risk of death and hospitalization among patients on hemodialysis (HD). We retrospectively evaluated the prevalence of mild cognitive impairment (MCI) in 421 patients on maintenance HD across nine facilities and investigated whether decreased handgrip strength was associated with decreased cognitive function. The Montreal Cognitive Assessment-Japan (MoCA-J) score and handgrip strength were measured. The mean age was 69.8 ± 11.2 years, and the median dialysis vintage 74.5 (IQR 30–150) months. The median MoCA-J score was 25 (IQR 21–27), and MCI was confirmed in 245 (58.2%) patients. Both the MoCA-J score and MoCA-J executive score were associated with age, history of cerebrovascular disease (CVA), and handgrip strength after adjustments. We found, among patients on HD aged under 70 years with a history of CVA, a handgrip strength < 90% (25.2 kg in males and 16.2 kg in females) correlated with significantly lower MoCA-J scores. A high prevalence of MCI and decreased handgrip strength were observed in patients on HD. Handgrip strength may be useful for the easy detection of MCI. A decrease in handgrip strength would allow for the early detection of MCI, especially among patients on HD aged under 70 years with a history of CVA.

## Introduction

Cognitive impairment is commonly observed in patients with chronic kidney disease (CKD)^1^. While silent brain infarcts and lacunar infarcts are well known to be associated with dementia^2^, we previously demonstrated that lacunar infarctions were more prevalent in patients with CKD, particularly those with decreased renal function and a creatinine clearance of < 40 mL/min/1.73m^2^^3^.

The brain and kidneys have many common anatomic and vasoregulatory features as they are low-resistance end organs exposed to high-volume blood flow, and susceptible to vascular damage^4^. Furthermore, nontraditional vascular risk factors—such as hyperhomocysteinemia, hypercoagulable states, inflammation, and oxidative stress—which are common in CKD, have been linked to cognitive impairment. We previously reported that hippocampal atrophy significantly correlated with hyperhomocysteinemia in patients on hemodialysis (HD)^5^, and demonstrated that regional cerebral blood flow decreased in all these patients, irrespective of their clinical symptoms or Mini-Mental State Examination (MMSE) scores^6^. Additionally, neurotoxicity is well known to be directly caused by uremic toxins^4^. A kidney-brain axis has been proposed based on many reports; therefore, it is plausible that the prevalence of cognitive dysfunction is higher in patients with CKD than in patients with normal kidney function^7^.

To identify dementia, it is essential to detect mild cognitive impairment (MCI) as early as possible. MCI is characterized by mild disorders in several key cognitive domains, including the executive functions of memory (learning and attention), problem solving (processing), and self-control (emotion-depression)^8^; it has been reported that dialysis initiation is associated with the loss of executive function, with no change in other aspects of cognition^9^. MCI is also considered a transitional stage, preceding clinical dementia. The Montreal Cognitive Assessment (MoCA) is a screening test for cognitive impairment that evaluates major cognitive domains—including episodic memory, language, attention, orientation, visuospatial ability, and executive functions,—while remaining brief and easy to administer^10^. A MoCA score ≤ 21 demonstrated a high predictive ability, sensitivity of 86%, and specificity of 55% for severe cognitive impairment, with a negative predictive value of 91%^11^. The reliability and validity of the Japanese version of the MoCA (MoCA-J) have been proven, and it has been recommended for geriatric health screening in the Japanese community^12^; using a cut-off point of 25/26, the MoCA-J demonstrated a sensitivity of 93.0% and specificity of 87.0% for screening MCI.

Frailty has also been associated with poor cognitive function in patients on HD^13^, while physical exercise has been shown to improve cognitive function in patients with end-stage kidney disease^14^. Handgrip strength is used as a reliable test to determine the functioning of skeletal muscle and is an independent predictor of all-cause mortality in patients on maintenance dialysis^15^; additionally, it can be easily measured. The relationship between MCI and physical function, including handgrip strength and gait speed, has been previously evaluated among older adults with predialysis CKD^16^; however, it was reported that gait speed, but not handgrip strength, was significantly associated with MCI. The aim of this study was therefore to evaluate the prevalence of MCI in patients on maintenance HD at multiple facilities across Japan, as well as to investigate whether decreased handgrip strength was associated with decreased cognitive function.

## Methods

### Study population

The protocol for this retrospective, cross-sectional, multicenter study was approved by the Tokushukai Group Institutional Review Board (TGE01229-024) and adhered to the tenets of the Declaration of Helsinki. Informed consent was obtained through an opt-out procedure; patients were provided with information explaining the proposed research project (purpose and required individual data) by means of an information sheet or a webpage on the hospital’s website and were given the opportunity to opt out.

The study assessed 465 patients on maintenance HD for > 3 months, as of 31 December 2016, at either of five HD clinics and four hospital-based outpatient units. Inclusion criteria were an age > 18 years and HD treatment three times per week, with no history of renal transplantation. Patients were excluded if they had any known neurological disorders, such as dementia or Parkinson’s disease; a history of mental illness, such as depression or schizophrenia; a history of delirium; any other severe health condition, such as malignancy or serious infection; or impairments in vision, hearing, language, or comprehension skills. Ultimately, 421 patients were included in this study.

### Data collection

Demographic, clinical, and laboratory data were retrieved from medical records. Demographic and clinical characteristics—including age, sex, weight, height, HD duration, smoking status (both current and former), prevalence of diabetes mellitus and hypertension, history of coronary artery disease and cerebrovascular disease (CVA ), and vascular access—were recorded along, with blood pressure, before starting HD. Laboratory data included serum albumin, calcium, phosphate, creatinine, urea nitrogen, C-reactive protein (CRP), intact parathyroid hormone, β2-microglobulin, total cholesterol, high-density lipoprotein cholesterol, and hemoglobin. Blood samples were routinely examined before the first HD session during the first week of December 2016.

### Cognitive assessment

Cognitive function was assessed using the MoCA-J^12^, a brief screening tool for MCI that evaluates multiple cognitive domains: memory, language, attention, visuospatial ability, orientation, concentration, executive function, conceptual thinking, and calculations. The MoCA-J scores ranged from 0 to 30, with a score < 26 suggestive of MCI. The MoCA is well known to be a more sensitive screening instrument for detecting cognitive impairment than the MMSE^12^. Additionally, the MoCA assesses executive function, a domain commonly affected in patients with CKD^10^. Cognitive function assessments were performed by an occupational therapist or nurse trained in using these assessments before initiating HD; to correct for education effects, one point was added to patients with an education level of ≤ 12 years.

### Measurement of handgrip strength

Handgrip strength was measured using a digital, standard adjustable-handle dynamometer (T.K.K.5401 GRIP D; Takei Scientific Instruments Co., Ltd., Niigata, Japan). Two measurements were obtained for each hand; an average value was calculated for each, and the higher value was adopted. Decreased handgrip strength was defined as < 28 kg in males and < 18 kg in females, based on the Asian Working Group for Sarcopenia^17^. To evaluate males and females concurrently, we also calculated the % handgrip strength corrected for 28 kg in males and 18 kg in females.

### Statistical analysis

Statistical analyses were performed using JMP version 13.2 (SAS Institute, Cary, North Carolina, United States). The data were expressed as mean (SD) for normally distributed continuous values (Kolmogorov–Smirnov test), and median (interquartile range [IQR]) for non-normally distributed continuous variables. Demographic and clinical data were presented according to the respective distribution type. Differences between groups were determined using an unpaired *t-*test for normally distributed values, and the Mann–Whitney *U-*test and c^2^ test, followed by Fisher’s exact test, for clinical characteristics, biochemical parameters, and handgrip strength.

Correlation coefficients were analyzed to establish the association with MCI, while multivariate linear regression analyses were used to clarify the relationship between MoCA-J scores and handgrip strength, clinical characteristics, and biochemical parameters; statistical significance was set at *p* < 0.05.

## Results

### Baseline characteristics

A total of 465 individuals on maintenance HD were screened for participation in the study. Forty-four patients had no handgrip strength data; thus, a total of 421 patients were finally analyzed. Table 1 shows the patients’ characteristics; the mean age was 69.8 ± 11.2 years, 64.8% were males, and all participants were Japanese. The median dialysis vintage was 74.5 (IQR 30–150) months. The number of patients with diabetes was 195 (46.3%), and 71 (16.9%) patients had a history of cerebrovascular accidents, including cerebral infarction and/or hemorrhage. One hundred ninety-eight participants (47%) were well educated, with a higher or university level of education.

**Table 1:** Patient characteristics.

Characteristic	Overall (n = 421)	Non-MCI (n = 176) MoCA-J ≥ 26	MCI (n = 245) MoCA-J < 26	*P* value
Age, [years]	69.8 ± 11.2	64.6 ± 10.4	73.6 ± 10.2	< 0.01
Male sex	273 (64.8)	106 (60.2)	167 (68.2)	0.1
BMI, [kg/m^2^]	21.0 [19.0–23.9]	21.5 [19.2–24.9]	20.6 [18.8–23.2]	0.02
Duration HD, [months]	74.5 [30–150]	82.5 [37–167]	70.5 [26–136]	0.03
Primary kidney disease				0.43
Diabetic nephropathy	141 (33.5)	55 (31.3)	84 (34.3)	
Chronic glomerulonephritis	128 (30.4)	57 (32.4)	73 (30.0)	
Nephrosclerosis	59 (14.0)	22 (12.5)	37 (15.1)	
Others or unknown	93 (22.1)	42 (23.9)	51 (20.8)	
Comorbidity				
Hypertension	269 (63.9)	111 (63.1)	158 (64.5)	0.36
Diabetes mellitus	195 (46.3)	79 (44.8)	116 (47.3)	0.31
Cerebrovascular disease	71(16.9)	21 (11.9)	50 (20.4)	0.01
Ischemic heart disease	43 (10.2)	13 (7.4)	30 (12.2)	0.08
Current smoker	55 (13.1)	30 (17.0)	25 (10.2)	0.17
Ultrafiltration volume [% BW]	4.34 ± 1.44	4.45 ± 1.33	4.27 ± 1.52	0.33
Systolic BP, [mmHg]	147 ± 23	148 ± 24	146 ± 23	0.44
Diastolic BP, [mmHg]	77 ± 13	80 ± 14	75 ± 13	< 0.01
Heart rate, [beat per minute]	76 ± 12	77 ± 12	75 ± 13	0.3
Hemoglobin, [g/dl]	10.6 ± 1.29	10.7 ± 1.10	10.6 ± 1.40	0.13
Albumin, [g/dl]	3.60 ± 0.34	3.68 ± 0.31	3.55 ± 0.35	< 0.01
CRP, [mg/dl]	0.14 [0.06–0.43]	0.11 [0.04–0.34]	0.16 [0.07–0.49]	0.01
Corrected calcium, [mg/dl]	8.85 ± 0.69	8.84 ± 0.74	8.86 ± 0.67	0.68
Phosphate, [mg/dl]	5.50 ± 1.42	5.54 ± 1.40	5.47 ± 1.43	0.69
Parathyroid hormone, [pg/ml]	187 [105–270]	164 [54–277]	198 [113–263]	0.15
β2-microglobulin, [mg/l]	26.9 ± 6.2	26.8 ± 6.1	27.0 ± 6.3	0.92
Educational level				0.55
< 12 years, n (%)	223 (53)	86 (49)	137 (56)	
> 12 years, n (%)	198 (47)	90 (51)	108 (44)	
MoCA-J, [points]	25 [21–27]	27 [26–29]	22 [20–24]	< 0.01
Handgrip strength, [kgf]	22.2 ± 8.4	24.5 ± 8.4	20.5 ± 8.1	< 0.01
Handgrip strength, [%]	90.1 ± 28.1	101.7 ± 25.9	81.7 ± 26.8	< 0.01

Values for categorical variables are presented as count (percentage); values for continuous variables are presented as mean ± standard deviation or median (interquartile range). The non-MCI group included 176 patients with normal cognitive function, defined as a MoCA-J score ≥ 26. The MCI group included 245 patients with mild cognitive impairment, defined as a MoCA-J score < 26.

Percent handgrip strength was calculated as follows: [males] handgrip strength [kg] × 100/28; [females] handgrip strength [kg] × 100/18.

Unpaired *t-tests*, Mann–Whitney *U-*tests, and c^2^ tests were used to evaluate differences.

*MCI* mild cognitive impairment, *MoCA-J* Japanese version of the Montreal Cognitive Assessment, *BMI* body mass index, *HD* hemodialysis, *BW* body weight, *BP* blood pressure, *CRP* C-reactive protein.

### MoCA-J score and difference between the MCI and non-MCI groups

**Figure 1:**
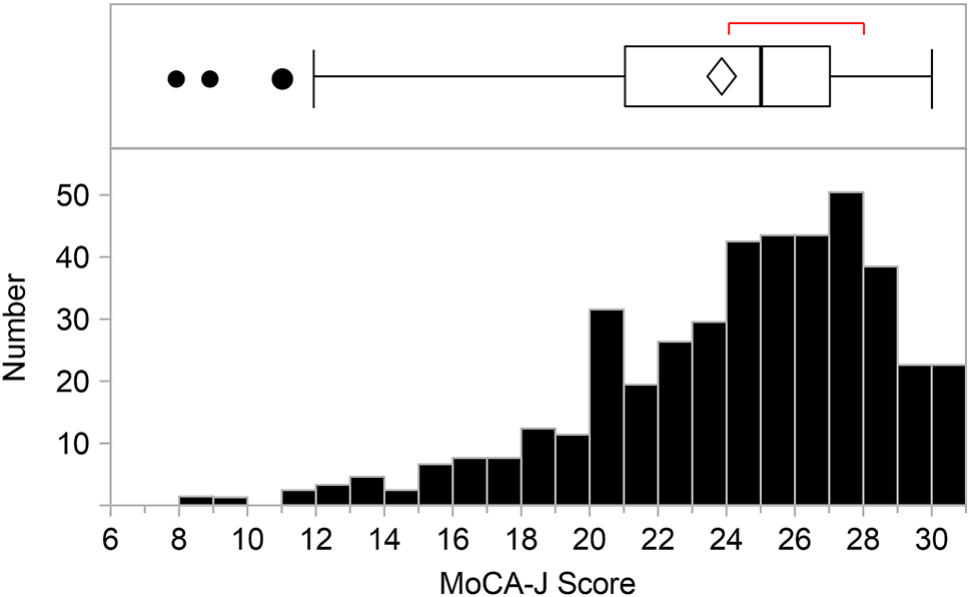
Montreal Cognitive Assessment-Japan (MoCA-J) score of patients on hemodialysis. The histogram illustrates the MoCA-J scores of the studied patients; of the 421 included patients, 58.2% (245 patients) had a MoCA-J score < 26.

The histogram of the MoCA-J scores ranged between 8 and 30 points (Fig. 1), with a median score of 25 (IQR 21–27; Table 1). A total of 145 (58.2%) patients were diagnosed with MCI, with a MoCA-J score < 26. Patients in the MCI group were significantly older, with a lower body mass index and shorter HD vintage (Table 1). Regarding comorbidities, the prevalence of CVAs was significantly higher in the MCI group; that of ischemic heart disease also tended to be higher in the MCI group. Patients with MCI were more likely to exhibit lower diastolic blood pressure (BP; 75 mmHg vs. 80 mmHg; *p* < 0.01). There were no differences regarding hemoglobin or serum β2-microglobulin levels between the MCI and non-MCI groups; however, serum albumin levels were significantly lower. Additionally, CRP levels were higher and handgrip strength lower in the MCI group.

### Handgrip strength

**Figure 2:**
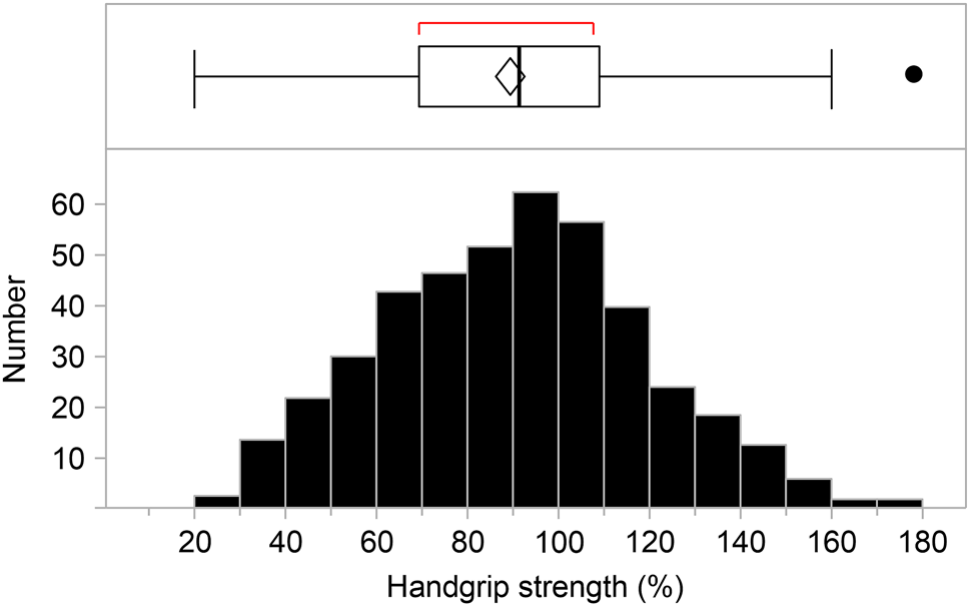
Handgrip strength of patients on hemodialysis. The histogram illustrates the handgrip strength of the studied patients. We defined 100% handgrip strength as 28 kg in males and 18 kg in females, according to the consensus report of the Asian Working Group for Sarcopenia (Chen LK, et al. *J Am Med Dir Assoc.* 2020;21:300–307). Of the 421 included patients, 63.1% (266 patients) demonstrated < 100% handgrip strength.

The average handgrip strength of all patients was 22.2 ± 8.4 kg (Table 1). We defined 100% of handgrip strength as 28 kg in males and 18 kg in females, according to the consensus report of the Asian Working Group for Sarcopenia^17^, as we wanted to evaluate both males and females at once. A handgrip strength < 100% met the sarcopenia standards; Fig. 2 includes a histogram demonstrating the handgrip strength of the participants. Of the 421 patients on HD, 63.1% (266 patients) demonstrated < 100% handgrip strength; i.e., over 60% of patients met the muscle strength criteria for sarcopenia (171 [62.3%] males and 95 [64%] females).

### Relationship between MoCA-J score, sex, and handgrip strength

**Figure 3:**
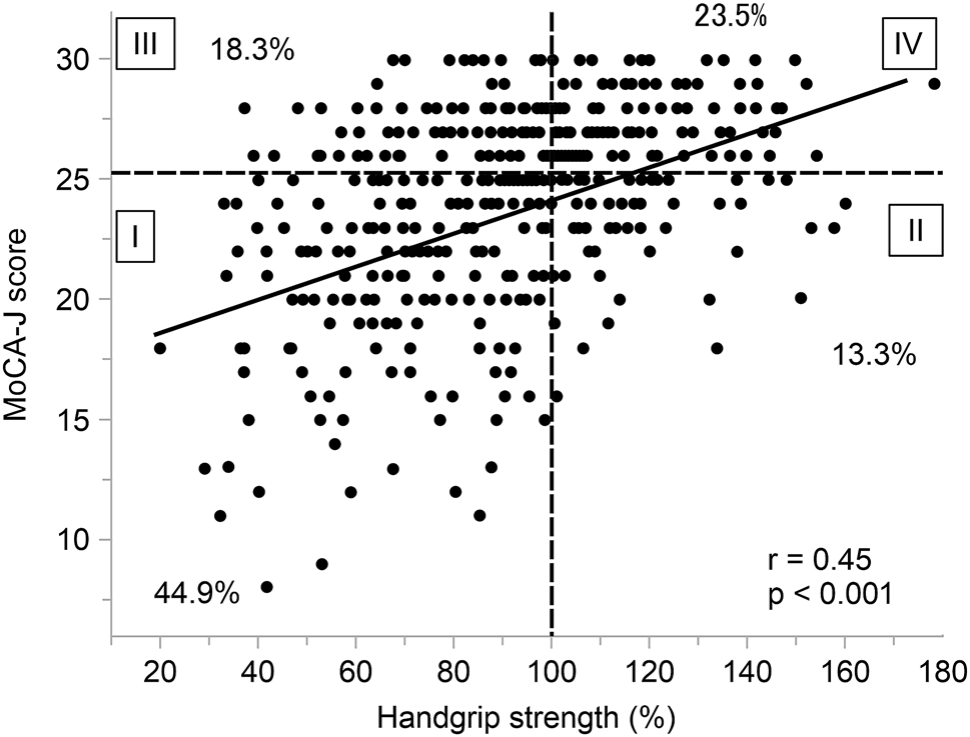
Relationship between Montreal Cognitive Assessment-Japan (MoCA-J) score and percent handgrip strength. The MoCA-J score demonstrated a significant positive correlation with handgrip strength (r = 0.45, *p* < 0.001). Dotted lines indicate the cutoff values of the MoCA-J score and percent handgrip strength.

**Figure 4:**
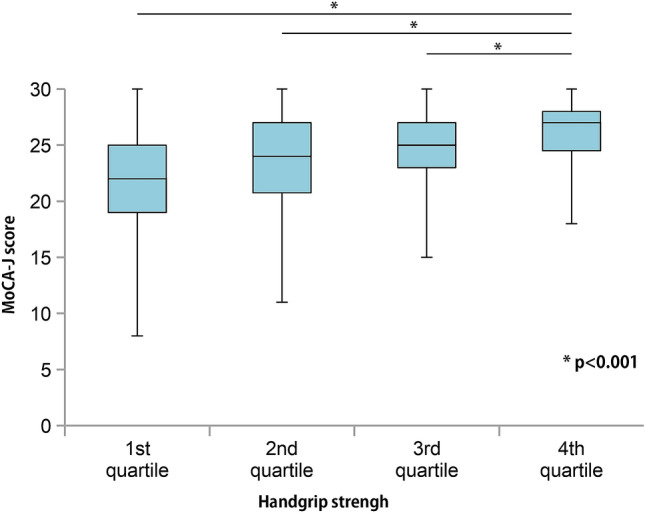
Montreal Cognitive Assessment-Japan (MoCA-J) scores for handgrip strength per quartile. Patients were divided into the following quartiles according to handgrip strength: first quartile [Q1], < 69.5% (19.5% in males and 12.5% in females); second quartile [Q2], ≥ 69.5% to < 91.0% (25.5 kg in males and 16.4% in females); third quartile [Q3], ≤ 91.0% to < 108.7% (30.4 kg in males and 19.6% in females); and fourth quartile (Q4), ≥ 108.7%. The median MoCA-J scores showed a significant upward trend from Q1 to Q4 (one-way ANOVA, *p* < 0.001). The MoCA-J score in Q4 was significantly higher than in the other quartiles (Tukey–Kramer post-hoc test; *p* < 0.001).

The MoCA-J scores were positively correlated with handgrip strength in both males and females (*p* < 0.001), while the MoCA-J score and percent handgrip strength were positively correlated (*r* = 0.45, *p* < 0.001; Fig. 3). A total of 189 (44.9%) patients had a MoCA-J score < 26 and percent handgrip strength < 100%, whereas 99 patients (23.5%) had a MoCA-J score ≥ 26 and percent handgrip strength ≥ 100%. Patients were divided into the following quartiles according to handgrip strength: first quartile (Q1), < 69.5% (19.5 kg for males and 12.5 kg for females); second quartile (Q2), ≥ 69.5% to < 91.0% (25.5 kg for males and 16.4 kg for females); third quartile (Q3), ≤ 91.0% to < 108.7% (30.4 kg for males and 19.6 kg for females); and fourth quartile (Q4), 108.7%. The median MoCA-J score showed a significant upward trend (one-way ANOVA, *p* < 0.0001; Fig. 4). The MoCA-J score of Q4 for handgrip strength was significantly higher than that of the other quartiles (Tukey–Kramer post-hoc test, *p* < 0.001; Fig. 4).

### Relationship between MoCA-J score and clinical characteristics

Results of the linear regression analysis are presented in Table 2. Correlation coefficients revealed that the MoCA-J score was associated with age, body mass index (BMI), cerebrovascular comorbidities, diastolic BP, serum albumin level, and handgrip strength. After adjusting for variables that demonstrated significant correlation coefficients, multivariate analysis revealed that the MoCA-J score was independently associated with age (coefficient β = − 0.33, 95% CI − 0.16 to − 0.09; *p* < 0.001), history of CVA (coefficient β = − 0.15, 95% CI − 1.30 to − 0.37; *p* < 0.001), and handgrip strength (coefficient β = 0.27, 95% CI 0.03–0.06; *p* < 0.001). As shown in Table 2, Figs. 3 and 4, handgrip weakness was an independent risk factor for a low MoCA-J score. A 10% increase (2.8 kg in males and 1.8 kg in females) in handgrip strength relates to a 27% increase in the MoCA-J score.

**Table 2:** Correlation coefficients and multivariate analyses of variables related to the MoCA-J score.

Variables included	Correlation coefficient (95% CI)	*P* value	Adjusted standardized partial regression coefficient β (95% CI)	*P* value
Age, per 1 year older	− 0.17 (− 0.20 to − 0.14)	< 0.001	− 0.12 (− 0.16 to − 0.09)	< 0.001
Male, vs female	Wilcoxon test	0.24		
BMI, per 1 kg/m^2^ increase	0.12 (0.02–0.21)	0.02	− 0.06 (− 0.15 to 0.03)	0.21
Duration of HD, per 1-month increase	0.0004 (− 0.004 to 0.005)	0.87		
CVA, vs without CVA	Wilcoxon test	< 0.001	− 0.83 (− 1.30 to − 0.37)	< 0.001
Diastolic BP, per 10 mmHg increase	0.51 (0.21–0.81)	< 0.001	− 0.08 (− 0.35 to 0.21)	0.59
Albumin, per 1 g/dL increase	2.62 (1.45–3.79)	< 0.001	0.77 (− 0.31 to 1.86)	0.16
CRP, per 1 mg/dL increase	− 0.02 (− 0.30 to 0.26)	0.89		
% handgrip strength, per 10% increase	0.66 (0.54–0.79)	< 0.001	0.40 (0.26–0.55)	< 0.001

Adjusted for all listed variables.

*MoCA-J* Japanese version of the Montreal Cognitive Assessment, *CI* confidence interval, *BMI* body mass index, *CVA* cerebrovascular disease, *CRP* C-reactive protein, *BP* blood pressure.

### Analysis stratified by age, with or without a history of CVA

Age, history of CVA, and handgrip weakness were independent explanatory factors for the MoCA-J score. JMP partition tree analysis between age and MCI demonstrated that an age of 69 was the best decision point; we therefore divided patients into two age groups: 70 years and over, and under 70 years. Furthermore, we divided patients according to history of CVA.

**Figure 5:**
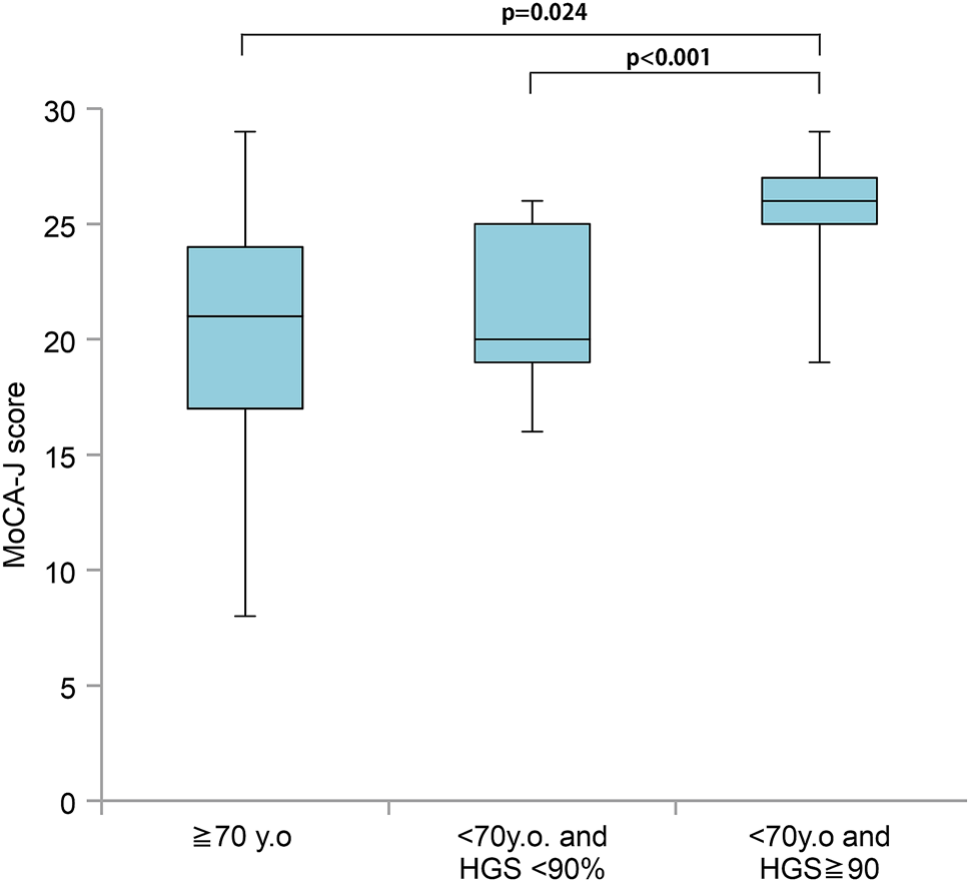
Montreal Cognitive Assessment-Japan (MoCA-J) scores among patients on hemodialysis with a history of cerebrovascular disease. Patients aged 70 years and over demonstrated significantly lower MoCA-J scores than patients aged under 70 years with a handgrip strength ≥ 90% (*p* = 0.024). Among those aged under 70 years with a history of cerebrovascular disease, those with a handgrip strength < 90% demonstrated significantly lower MoCA-J scores than those with a handgrip strength ≥ 90% (*p* < 0.001).

We investigated the relationship between handgrip strength and MoCA-J scores in patients on HD aged under 70 years with a history of CVA. Predictive values of handgrip strength for MCI were analyzed using receiver operating characteristic (ROC) analysis; the best predictor of MCI was handgrip strength (AUC = 0.713), with an optimal cut-off point of 90% (Youden Index = 0.353). Figure 5 shows the MoCA-J scores of the patients on HD with a history of CVA; they were divided into three age groups: 70 years and over, under 70 years with a handgrip strength < 90%, and under 70 years with a handgrip strength ≥ 90%. We found that among patients on HD aged under 70 years with a history of CVA, a handgrip strength < 90% (25.2 kg for males and 16.2 kg for females) was associated with significantly lower MoCA-J scores. Conversely, no such relationship was found between grip strength and MoCA-J score among patients on HD with no history of CVA (data not shown).

### Relationship between the MoCA-J executive score and clinical characteristics

A previous study demonstrated that the initiation of dialysis was associated with a loss of executive function, with no change in other aspects of cognition^9^, we focused on the executive score. The MoCA-J executive score also correlated with age (coefficient β = − 0.13, *p* = 0.013), history of CVA (coefficient β = − 0.23, *p* < 0.001), and handgrip strength (coefficient β = 0.095, *p* < 0.001) after adjusting for the covariates in the correlation coefficients (Table 3). Handgrip strength was also significantly correlated with MoCA-J executive function.

**Table 3:** Correlation coefficients and multivariate analyses of variables related to the MoCA-J executive score.

Variables included	Correlation coefficient (95% CI)	*P* value	Adjusted standardized partial regression coefficient β (95% CI)	*P* value
Age, per 1 year older	− 0.025 (− 0.034 to − 0.016)	< 0.001	− 0.013 (− 0.024 to − 0.003)	< 0.001
Male, vs female	Wilcoxon test	0.37		
BMI, per 1 kg/m^2^ increase	0.013 (− 0.013 to 0.038)	0.33		
Duration of HD, per 1-month increase	− 0.001 (− 0.002 to 0.001)	0.31		
CVA, vs without CVA	Wilcoxon test	< 0.001	− 0.23 (− 0.37 to − 0.10)	< 0.001
Diastolic BP, per 10 mmHg increase	0.003 (− 0.01 to 0.06)	0.78		
Albumin, per 1 g/dL increase	0.29 (− 0.08 to 0.60)	< 0.066		
CRP, per 1 mg/dL increase	− 0.01 (− 0.30 to 0.26)	0.89		
% handgrip strength, per 10% increase	0.13 (0.09–0.16)	< 0.001	0.10 (− 0.05 to − 0.13)	< 0.001

Adjusted for all listed variables.

*MoCA-J* Japanese version of the Montreal Cognitive Assessment, *CI* confidence interval, *BMI* body mass index, *CVA* cerebrovascular disease; *CRP* C-reactive protein, *BP* blood pressure.

## Discussion

The aim of this cross-sectional, multi-institutional study was to evaluate the prevalence of MCI in patients on maintenance HD in multiple facilities across Japan, as well as the association between MCI and muscle weakness in these patients. Our results revealed that of 421 patients on dialysis, 58.2% were diagnosed with MCI according to the MoCA-J score. The MoCA-J score, including executive function score, was significantly associated with handgrip strength.

In 2018, the average age and dialysis vintage of Japanese patients on dialysis was 68.7 years and 88.6 months, respectively^18^; therefore, the study cohort was considered to represent the current situation of dialysis patients in Japan. The prevalence of MCI in our study was 58.2%; conversely, a recent, prospective, observational cohort study reported that cognitive impairment was present in 43 (48.9%) participants on HD—who were not known to have cognitive impairment—based on a MoCA score < 26^19^. The median age of the subjects was 58 years, over 10 years younger than our cohort, suggesting a lower prevalence of MCI than observed in our study; more than half of the patients in our study were diagnosed with MCI. Since MCI is a transitional stage preceding clinical dementia, with cognitive impairment exceeding normal aging and age-associated decline^8^, it is necessary to detect MCI early and take appropriate interventional action. Petersen et al. established the concept of MCI first from the observation of patients with MCI, patients with mild Alzheimer disease and healthy control subjects^20^. However, MCI, as described in this paper, is not related to Alzheimer disease. According to “Clinical Practice Guideline for Dementia 2017”, edited by Japanese Society of Neurology, MCI is a diagnosis based on symptoms, and the pathological background is diverse^21^.

Sarcopenia—a chronic condition associated with aging and characterized by a reduction in muscle mass, strength, and function^17^—was reported to affect approximately 37% of patients on dialysis^22^. The loss of muscle mass correlates with greater morbidity and mortality, particularly due to an increase in cardiovascular complications^23^. Diagnosing sarcopenia requires measurement of both muscle quality and quantity. Low muscle strength (handgrip strength < 28 kg in males and < 18 kg in females), irrespective of reduced physical performance (five Times Sit-to-Stand Test ≥ 12 s) was defined as “possible sarcopenia”^17^; 63.1% (266 patients) of the patients in our study were thus considered to have “possible sarcopenia”.

We demonstrated that age, cerebrovascular comorbidities, and handgrip strength were significantly associated with MCI. Regarding the relationship between cognitive impairment and sarcopenia, a systematic review demonstrated that the odds ratio for cognitive impairment in patients with sarcopenia (not only kidney diseases), compared with patients without sarcopenia, was 2.25 (95% CI 1.70–2.97) in the adjusted meta-analysis^24^. The etiology of uremic sarcopenia is thought to increase proinflammatory cytokines, as well as decrease protein intake, exercise, sex hormones, growth hormones, insulin, vitamin D, and the number of satellite cells^23^. With respect to cognitive function, there are many overlapping mechanisms, such as the increase in proinflammatory cytokines, effects of uremic toxins, and etiology of sarcopenia^4^.

The MoCA is thought to be a suitable tool for assessing cognitive function in patients on HD, as it covers executive functions^10^; loss of executive function, with no change in other aspects of cognition, is a distinctive feature in these patients^9^. Executive function was evaluated via the results of the trail making test (part B), cube copying, and clock drawing, with 5 points indicating a perfect score. We performed linear regression analysis to clarify the relationship between the MoCA-J scores for executive function and various parameters; the MoCA-J executive score was found to be significantly associated with age, history of CVA, and grip strength after adjusting for variables.

We found that handgrip strength was particularly useful for predicting MCI in patients on HD aged under 70 years, with a history of CVA. A handgrip strength < 90% (males: 25.2 kg; females: 16.2 kg) demonstrated significantly low MoCA-J scores; therefore, we should examine the cognitive function of patients on HD aged under 70 years with a history of CVA if they exhibit decreased handgrip strength. In other words, the MoCA-J test is a tedious and time-consuming alternative by comparison. The present study demonstrates that the MoCA-J test is significantly correlated with handgrip strength, which is easily measured in the dialysis unit, and possible in repeated measurements. Measurement of handgrip strength is a simple, quick, noninvasive, inexpensive, and objective procedure; therefore, assessment may help to screen for the early stages of MCI.

This study had several limitations; first, we only used the MoCA-J to assess the MCI. We excluded patients with apparent depressive HD; however, depression may affect cognitive impairment^8^, and the coexistence of depression and MCI might be a more general phenomenon—rather than two different nosological entities—in patients on HD. Second, intradialytic cerebral hypoperfusion is considered one of the mechanisms of cognitive impairment in patients on HD^25^; unfortunately, however, the frequency of intradialytic hypotension was not measured in this study. Indeed, a recent study regarding the relationship between hypotension and cerebral ischemia during HD demonstrated that BP poorly predicted downstream cerebral ischemia^26^. They concluded that intradialytic cerebral ischemia, but not hypotension, correlated with decreased executive cognitive function. Research from the perspective of intradialytic cerebral hypoperfusion should thus be conducted in the future.

## Conclusions

A high prevalence of MCI and decreased handgrip strength were observed in patients on HD. Since age, cerebrovascular comorbidities, and handgrip strength were found to be associated with MCI, handgrip strength may be useful for the easy detection of MCI; therefore, a decrease in handgrip strength would allow for the early identification of impaired cognitive function, particularly in younger patients on HD with a history of CVA.

## Data Availability

The datasets used and/or analyzed during the current study available from the corresponding author on reasonable request.
